# Distinctive Structural and Effective Connectivity Changes of Semantic Cognition Network across Left and Right Mesial Temporal Lobe Epilepsy Patients

**DOI:** 10.1155/2016/8583420

**Published:** 2016-11-29

**Authors:** Xiaotong Fan, Hao Yan, Yi Shan, Kun Shang, Xiaocui Wang, Peipei Wang, Yongzhi Shan, Jie Lu, Guoguang Zhao

**Affiliations:** ^1^Department of Neurosurgery, Xuanwu Hospital, Capital Medical University, Beijing 100053, China; ^2^Departments of Psychology and Linguistics, Xidian University, Xi'an 710126, China; ^3^Neuroimaging Laboratory, School of Biomedical Engineering, Shenzhen University Health Science Center, Shenzhen 518060, China; ^4^Department of Radiology, Xuanwu Hospital, Capital Medical University, Beijing 100053, China; ^5^Department of Nuclear Medicine, Xuanwu Hospital, Capital Medical University, Beijing 100053, China; ^6^Department of Biomedical Engineering, School of Life Science and Technology, Xi'an Jiaotong University, Xi'an 710049, China; ^7^Center of Stroke, Beijing Institute for Brain Disorder, Beijing 100069, China; ^8^Beijing Key Laboratory of Magnetic Resonance Imaging and Brain Informatics, Beijing 100053, China; ^9^Center of Epilepsy, Beijing Institute for Brain Disorder, Beijing 100069, China

## Abstract

Occurrence of language impairment in mesial temporal lobe epilepsy (mTLE) patients is common and left mTLE patients always exhibit a primary problem with access to names. To explore different neuropsychological profiles between left and right mTLE patients, the study investigated both structural and effective functional connectivity changes within the semantic cognition network between these two groups and those from normal controls. We found that gray matter atrophy of left mTLE patients was more severe than that of right mTLE patients in the whole brain and especially within the semantic cognition network in their contralateral hemisphere. It suggested that seizure attacks were rather targeted than random for patients with hippocampal sclerosis (HS) in the dominant hemisphere. Functional connectivity analysis during resting state fMRI revealed that subregions of the anterior temporal lobe (ATL) in the left HS patients were no longer effectively connected. Further, we found that, unlike in right HS patients, increased causal linking between ipsilateral regions in the left HS epilepsy patients cannot make up for their decreased contralateral interaction. It suggested that weakened contralateral connection and disrupted effective interaction between subregions of the unitary, transmodal hub of the ATL may be the primary cause of anomia in the left HS patients.

## 1. Introduction

Temporal lobe epilepsy (TLE) is the most common drug resistant epilepsy in adults. The majority of seizures in TLE are associated with hippocampal sclerosis (HS) or other temporal lobe abnormalities [1], which can reliably be detected in vivo by MRI [2, 3]. Patients with resection for TLE generally do not report comprehension difficulties through either clinical reports or formal testing [4] but complain of significant amnesia and anomia which reflect a semantic weakness [5–7]. A systematic review calculating pooled estimates of neuropsychological outcomes reported a 44% risk of decline in verbal memory and 34% risk of decline in naming after left-sided surgery [8]. But there are no reports of naming decline following nondominant hemisphere resection [9]. It reflects that left and right HS patients may experience distinctive functional reorganization in the nonepileptic temporal lobe under distinctive compensatory mechanisms to sustain key cognitive functions, such as language.

Semantic cognition can be decomposed into three interactive principal components implemented by separable neural networks: (1) semantic entry/exit or conceptualization (translation between sensation/motor representations and semantic knowledge); (2) the long-term representation of concepts/semantic memory; and (3) semantic control (mechanisms that interact with our vast quantity of semantic knowledge in order to generate time- and context-appropriate behavior) [10, 11]. By means of activation likelihood estimate (ALE) technique, Binder et al. reported a distinct, left-lateralized network specialized for storage and retrieval of semantic knowledge [12]. The related areas included posterior inferior parietal lobe (angular gyrus, AG; supramarginal gyrus, SMG), middle temporal gyrus (MTG), fusiform, parahippocampal gyrus, dorsomedial prefrontal cortex (dmPFC), inferior frontal gyrus (IFG), ventromedial prefrontal cortex (vmPFC), and posterior cingulate cortex (PCC). It is proposed that concepts are formed through the convergence of sensory, motor, and verbal experience via the left anterior temporal lobe (ATL), a transmodal representational hub [13] which primarily links pertinent semantic/conceptual information into the language system to produce a specific name [14]. The conclusion that the ATL is a crucial component in semantic cognition has been bolstered by contemporary basic neuroscience studies utilizing magnetoencephalography, distortion-corrected functional MRI, PET, or repetitive transcranial magnetic stimulation [15]. Meanwhile, the claim that posterior temporoparietal areas are associated with semantic control has been demonstrated in both semantic aphasia patients and healthy people [11, 16].

Concerning the common phenomenon that left HS patients, especially those after surgical resection of ATL, can perform within the normal accuracy range on standard semantic assessments but show measureable anomia [6, 17–20], we posited that a semantic-lexical disruption in the intermediate processing step that relayed retrieved semantic information on to the language system resulted in the primary problem in naming. Since temporal lobe epilepsy is considered as a network disease [21], its pathological feature requires us to examine the abnormal function of a whole network rather than a single epileptogenic region. To explore dysfunction of neural networks, functional connectivity changes in epilepsy patients have been tested by different neuroimaging modalities, such as repetitive transcranial magnetic stimulation (rTMS) [22], corticocortical evoked potentials (CCEP) [23], and EEG [24]. Although some of these techniques have the noninvasive advantage, resting state fMRI (rsfMRI) analysis possesses additional gains: resting state networks (RSNs) are highly organized in space, are reproducible from subject to subject, and differ with aging and between genders [25]. In addition, it also allows the search for significant baseline fluctuations to obtain task-free functional network information and identify epileptic circuits by providing clinicians and neurosurgeons with clues about where new or secondary epileptic foci may form, where seizures place most burden on the brain, and where are new core functional regions.

fMRI functional connectivity describes brain function and cooperation at a network level by identifying regions that make up a network of interest. It reflects the degree of signal synchrony between anatomically distant brain regions during resting state or tasks. However, these linear correlations do not provide information on the direction of influence between regions. Coefficient-based GCA is a directed functional connectivity method [26]. Given that imbalance of excitatory and inhibitory effect is a fundamental change in epilepsy [27], the GCA technique has a special advantage for investigating the pathophysiological mechanism of HS patients by means of quantifying the magnitude and direction of influence of one region time series on another [28, 29].

In sum, previous studies reported that left and right HS patients are differently impaired in semantic cognition. These patients offer the opportunity to study different impacts of focal structural lesions on functional connectivity within the semantic network. The objectives of this study were (1) to evaluate and contrast the occurrence of gray matter (GM) atrophy in patients with left and right HS in the semantic cognition network and (2) to quantify direction of influence between these anatomical regions using Granger causality analysis. We hypothesized that the presence of HS is consistent with more pronounced, diffuse GM atrophy with the semantic atrophy the most severely damaged; left HS patients' classical anomia (i.e., can provide good information about unnamed items) were caused by the unique deficit pattern of disrupted functional connectivity of the left anterior superior temporal lobe and other regions underlying semantic memory.

## 2. Methods

### 2.1. Participants

Twenty-four right-handed TLE patients (17 females, 7 males; age range 16–48 years; mean age 29.00 ± 9.57 years; epilepsy onset 12.46 ± 9.06 years; epilepsy durations 15.86 ± 7.43 years) with unilateral HS (13 left HS and 11 right HS) were recruited from Xuanwu Hospital Capital Medical University. All patients underwent a comprehensive clinical evaluation and fulfilled the following inclusion criteria: (1) typical symptoms of TLE as complex partial seizures, accompanied, or not, by simple partial seizures; some patients had aurae, like epigastric rising, hallucination, and so on; the seizure frequency was 4-5 times per day at most and 1 time per month at least; (2) standard MRI criteria for HS (hippocampal atrophy, increased T2 signal, and loss of internal hippocampal architecture) which were finally confirmed by histopathology; (3) typical EEG findings (interictal spike or sharp waves at the anterior temporal area in both wakefulness and/or sleep, various ictal rhythms including background attenuation, start-stop-start phenomenon, irregular 2–5 Hz lateralized activity, and 5–10 Hz sinusoidal waves or repetitive epileptiform discharges) [30]; (4) no other neuropsychopathic diseases like intracranial tumor, cerebral hemorrhage, infarction, trauma, schizophrenia, affective psychosis, and so on. The clinical and demographic data of all patients were shown in Table 1. Healthy adult controls (HC) without neurological or psychiatric medical history or medication known to impair memory were recruited. HC group consisted of 24 age and gender matched healthy controls (17 females, 7 males; mean age 29.50 ± 10.18 years). There was no difference between the three groups of left HS, right HS, and normal controls (*F* = 0.499, *p* = 0.611). The local Ethics Committee approved the study and all participants gave written informed consent according to the Declaration of Helsinki prior to the study.

### 2.2. MRI Data Acquisition

MRI images were acquired during interictal stage with a 3.0 T scanner (MAGNETOM Tim Trio, Siemens Healthcare, Erlangen, Germany) using the 12-channel phased-array head coil supplied by the vendor. Structural images were acquired with a sagittal MP-RAGE three-dimensional T1-weighted sequence (TR = 1600 ms, TE = 2.15 ms, flip angle = 9°, thickness = 1.0 mm, and FOV = 256 mm × 256 mm). Functional images were acquired using the gradient echo-planar pulse sequence (TR = 3000 ms, TE = 30 ms, flip angle = 90°, and thickness = 3 mm). Participants were instructed to stay awake with eyes closed.

### 2.3. Regions of Interest (ROIs) Identification

In accordance with previous studies mentioned in the introduction part, all ROIs were defined in accordance with the AAL template, such as temporal pole of superior temporal gyrus (tpSTG) and temporal pole of middle temporal gyrus (tpMTG) that functions in conceptualization; MTG, fusiform, parahippocampal gyrus, dmPFC (medial superior frontal gyrus in AAL template), IFG, vmPFC (medial orbitofrontal gyrus in AAL template), and PCC that functions in memory storage; angular gyrus (AG) and supramarginal gyrus (SMG) that function in semantic control. Given that GM volume decrease has been reported in both ipsilateral and contralateral temporal neocortex [31–33], and mTLE patients always show atypical language lateralization [34], the current study selected 12 ROIs in each hemisphere (24 ROIs in all).

### 2.4. Structural Analysis

Volumetric data for cortical and subcortical structures were analyzed by optimized VBM and FIRST, parts of FSL tools, separately. The principal focus of the current study was to contrast different structural damages to the semantic cognition network in both the left and right HS patients. To make sure the severe damage to semantic cognition network was not a by-product of overall gray matter volume (GMV) loss, we also quantified structural changes of all regions between patients and controls.

The initial stages of VBM analysis included removing nonbrain tissues by Brain Extraction Tool and tissue-type segmentation with FAST4. The resulting GM partial images were then aligned to the MNI 152 template by affine-registration. A symmetric study-specific GM template was created by averaging images and flipping along the *x*-axis. Next, all the GM images which were nonlinearly registered to the study-specific GM template were modulated and smoothed by Gaussian kernels with a sigma of 3 mm. Regions within the semantic cognition network from WFU atlas were selected as ROIs in further two-sample* t*-test by randomization (5000 permutations) with TFCE implemented, between controls and left or right HS subgroups, respectively. In order to rule out the possibility that group difference was caused by the different pattern of whole brain atrophy among left and right HS patients, we added the remaining ROIs from WFU atlas in additional analyses to examine volumetric changes as aforementioned. All the volumetric results were considered statistically significant after FWE-correction at *p* < 0.05, with cluster including more than 10 continuous voxels.

FIRST was used to segment the subcortical structures, including bilateral thalamus, hippocampus, amygdala, caudate nucleus, putamen, and globus palladium. The left and right mean volume of these nucleus were extracted with fslstats. We calculate the normalized volume of subcortical structures by multiplying the scaling factor obtained from SIENAX.

### 2.5. Granger Causality Analysis

Functional preprocessing steps were carried out using the statistical parametric mapping (SPM5). It included the following steps: (1) slice timing correction; (2) trilinear sinc interpolation for alignment (motion correction); (3) spatial normalization based on the MNI space and resampled at 3 mm × 3 mm × 3 mm; (4) band-pass filter (0.01~0.08 Hz) spatially smoothed with a 6 mm full-width-at-half maximum (FWHM) Gaussian kernel; and (5) head motion and ventricular and white matter signal regression.

The multivariate GCA (mGCA) has been proved to be an optimal candidate to investigate the causal networks for its data-driven nature [35]. We performed mGCA on the time series of BOLD signal intensities from selected ROIs in both groups. The entire time series were averaged across voxels within each ROI picked in each group and were then normalized across subjects to form a single vector per ROI. The mGCA detected causal interactions by computing directed transfer function (DTF) from a multivariate autoregressive model of the time series [36]. We also adopted weighted DTF with partial coherence in order to emphasize direct connections and deemphasize mediated influences [36, 37]. The statistical significance of the path weights was ascertained using surrogate data. Surrogate data were generated by randomizing the phase of the original time series spectrum while retaining its magnitude. A null distribution was obtained by generating 2500 sets of surrogate data and calculating the direct directed transfer function (dDTF) from these 2500 datasets. The dDTF value obtained from the original time series was verified using a null distribution for the one-tailed test with the significant *p* value of 0.01. In addition, a difference of influence (doi) term was used to assess links that showed a dominant direction of influence [38], which limits potentially spurious links caused by hemodynamic blurring [29]. The effective connectivity network of the 9 ROIs was constructed by visualizing the significant dDTF (*p* < 0.01, FDR corrected for multiple comparisons) obtained after running the statistical significant test.

The high degree nodes were considered to be the hubs of network [39]. We calculated “in-degree” (number of Granger causal efferent connections to a node) to find the central targets of network, and “out-degree” (number of Granger causal afferent connections from a node) to find the central sources [40, 41]. Further, hubs of the network were defined if the sum of “in-degree” and “out-degree” of a node was at least 1.96 standard deviations (SD) greater than the average of “in- + out-degree” of all nodes in the semantic cognition network [42].

Between-group differences in the causal connectivity graphs were determined as follows. We calculated dDTF values in all connections for every subject to explore the difference in the intensity of effective connectivity between groups, particularly with volumetric value of relevant ROI as a regressor. The links that showed between-group changes in the strength of causal influence were those whose difference in the doi term significantly differed between groups by a paired* t*-test (*p* < 0.05, FDR corrected).

### 2.6. Statistical Analysis

Analyses were performed using SPSS (IBM Corp. Released 2012. IBM SPSS Statistics, Version 21.0, Armonk, NY, USA). Volumetric comparison for normalized subcortical structures was conducted by two-sample* t*-test (*p* < 0.05). For each participant, a laterality index (LI) of hippocampus volume (HV) was computed using the formula [(left HV − right HV)/(left HV + right HV)]. A one-way ANOVA was used to compare LIs of left HS, right HS, and controls. Pearson's correlations were then used to examine the relationships between LI and factors such as age at epilepsy diagnosis, years since diagnosis, seizure frequency, and linking strength between ROIs in each group.

## 3. Results

### 3.1. Anatomical Changes

**Figure 1:**
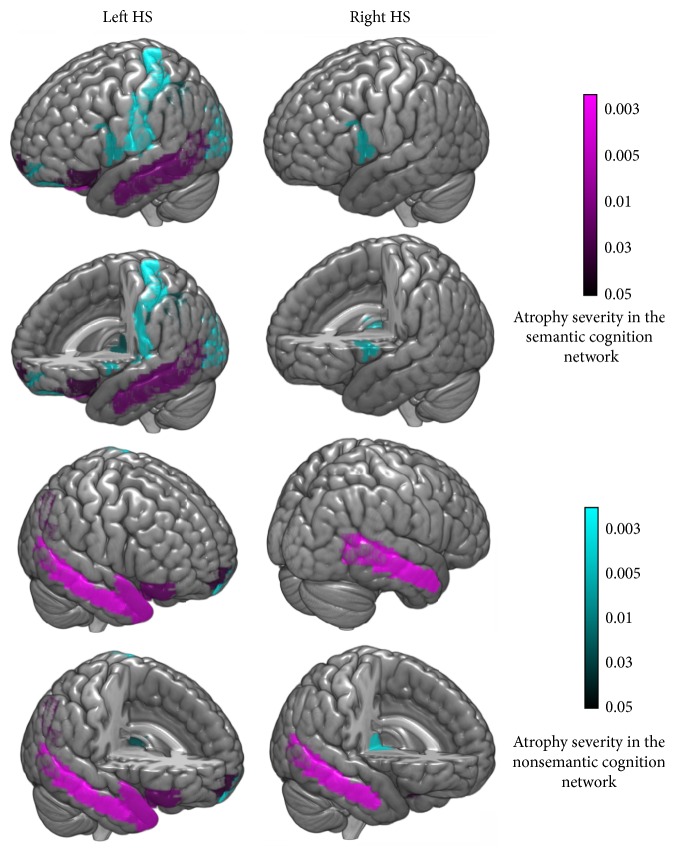
Gray matter volume (GMV) loss of patients with left HS or right HS (FWE corrected, *p* = 0.05; minimum cluster size 10). In the left HS subgroup, GMV loss was observed in 12 ROIs in the ipsilateral lobe (including the IFG, vmPFC, hippocampus, and MTG in the semantic cognition network) and 7 ROIs in the contralateral lobe (including the IFG, vmPFC, parahippocampus, fusiform, AG, tpSTG, tpMTG, and MTG, all of which were components of the semantic cognition network); in the right HS subgroup, GMV loss was seen in 2 ROIs in the ipsilateral lobe and the MTG in the contralateral lobe.

Group comparisons between controls and patients with left or right HS identified GMV loss confined to bilateral hemispheres (FWE corrected, *p* ≤ 0.05). Specifically, in the left HS subgroup, GMV loss was found in 12 ipsilateral ROIs and 7 contralateral ROIs (Figure 1, left column); in the right HS subgroup, GMV loss was in 2 ipsilateral ROIs and 1 contralateral ROI (Figure 1, right column). Within the semantic cognition network, left HS patients showed atrophy in 4 areas of IFG, vmPFC, hippocampus, and MTG in the ipsilateral lobe, and 7 areas of IFG, vmPFC, parahippocampus, fusiform, AG, tpSTG, tpMTG, and MTG in the contralateral lobe. Meanwhile, right HS patients showed only ipsilateral MTG atrophy and no contralateral atrophy (see details in Table 2).

**Figure 2:**
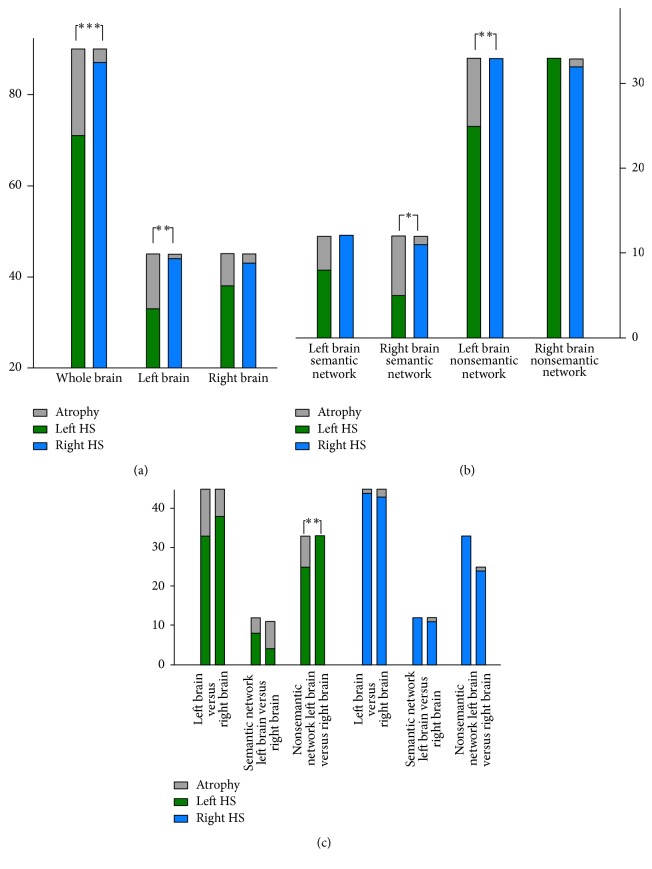
Comparison of atrophy severity of the left and right HS patients in both hemispheres. (i) The whole brain and especially the left hemisphere were more severely damaged in left HS patients than right HS patients. (ii) The atrophy severity of the semantic cognition network in the right hemisphere was more severe for left HS patients than right HS patients. (iii) “*∗*” indicates *p* < 0.05; “*∗∗*” indicates *p* < 0.01; “*∗∗∗*” indicates *p* < 0.005.

Further analysis showed significant difference of atrophy severity in the left and right HS patients (19/90 : 3/90; atrophy area number/network number; *p* < 0.001). In particular, the left hemisphere was more severely damaged in the left HS patients than the right ones (12/45 : 1/45; *p* < 0.005) (Figure 2(a)). In addition, atrophy severity of the semantic cognition network in the right hemisphere was more severe in the left HS patients than the right ones (7/12 : 1/12; *p* < 0.05), and patients' atrophy difference of the left nonsemantic cognition network also reached a significant level (8/33 : 0/33; *p* < 0.01) (Figure 2(b)). Even though, in the left HS patients, there was no significant differences of atrophy between left and right hemispheres (12/45 : 7/45; *p* = 0.20) or between the left and right semantic cognition network (4/12 : 7/12; *p* = 0.22), their nonsemantic networks were more severely damaged in the left hemispheres (8/33 : 0/33; *p* < 0.01) (Figure 2(c)).

### 3.2. Structural Asymmetry

A one-way ANOVA revealed significant differences between groups for hippocampal laterality index (*F* = 12.70, *p* < 0.001). The LI was highest (most left-lateralized) in the right HS patients (mean = 0.13, SD = 0.05), followed by the healthy controls (mean = −0.01, SD = 0.03) and the left HS group (mean = −0.21, SD = 0.05). Subsequent contrasts revealed significant differences between controls and patients with left HS (*t* = 3.31, *p* = 0.002), between controls and right HS patients (*t* = −2.80, *p* < 0.01), and between groups of left and right HS (*t* = −4.27, *p* < 0.001).

### 3.3. Strength Changes of Functional Connectivity in Left and Right HS Patients

**Figure 3:**
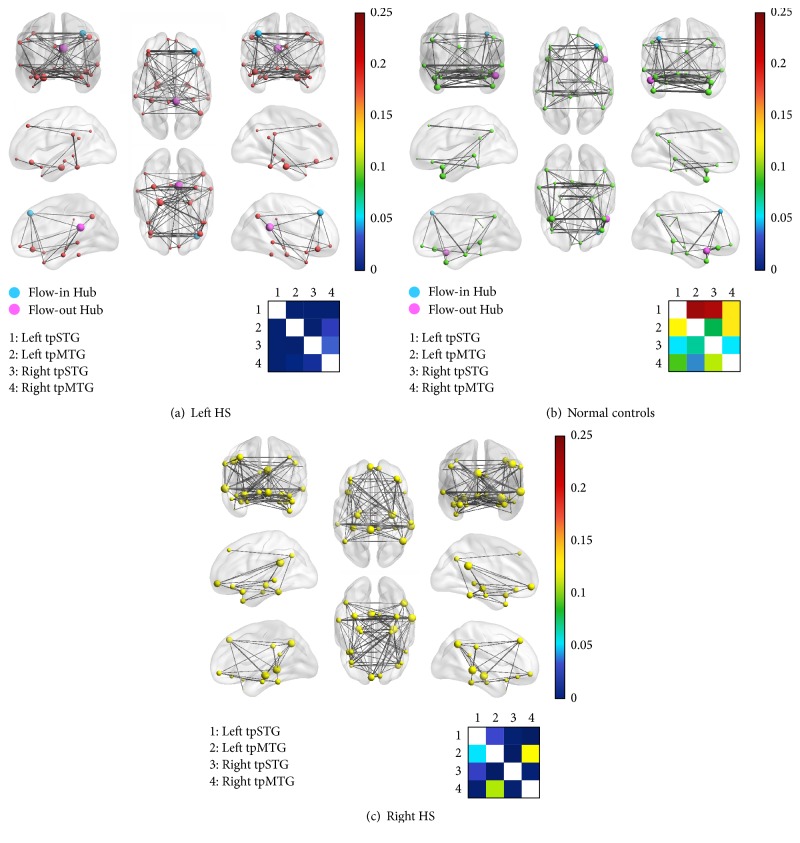
Causal influence of effective connectivity between ROIs in the semantic cognition network. (i) Connection density among the three groups of left HS patients, right HS patients, and normal controls was not significantly different from each other. (ii) The network hub in the normal controls was the right tpMTG (the flow-in hub the right dmPFC and the flow-out hub the right tpMTG), and the network hub in the left HS patients was the right PCC (the flow-in hub the right dmPFC and the flow-out hub the right PCC), but there was no hub node in the semantic cognition network of patients with right HS. (iii) Subregions of the anterior temporal lobe (ATL) in the bilateral hemispheres were strongly connected in normal controls but partially interconnected in right HS patients and not interconnected in left HS patients.

A causal connectivity graph was constructed using the thickness of connecting lines to indicate the strengths of causal influences (see Figure 3). For left HS patients, right HS patients, and healthy controls, causal influences within the semantic cognition network presented strongly covarying relations (Figures 3(a), 3(b), and 3(c)). Overall, connection density among the three groups was not significantly different (*F* = 0.03, *p* = 0.97). But interconnection patterns between ATL subregions were different in the three groups: all the four subregions (left/right tpSTG and tpMTG) were significantly connected with each other in normal controls; no causal influence of the areas was found in the left HS patients; little causal influence remained in the right HS patients (colored check-boards at the bottom of each part in Figure 3).

Meanwhile, node degree analysis yielded more differences between groups. In the normal controls, the only hub of semantic cognition network was the right tpMTG. Specifically, the flow-in hub was the right dmPFC, while the right tpMTG was the only flow-out hub. In the left HS patients, the only hub was the right PCC. Specifically, the flow-in hub was the right dmPFC, while the right PCC was the only flow-out hub. However, there was no hub node in the semantic cognition network of right HS patients. Further, by comparing node degree between the left HS, right HS, and controls, we found that node degree of the right PCC was significantly different between left HS patients and controls (*t* = −2.23, *p* < 0.05), and node degree of the right MTG was significantly different between right HS patients and controls (*t* = 3.42, *p* < 0.002). By comparing in-degree and out-degree between the left HS and controls, we found that the out-degree of right PCC (*t* = −2.25, *p* < 0.05), right IFG (*t* = 2.67, *p* < 0.02), left MTG (*t* = 2.20, *p* < 0.05), and left tpMTG (*t* = −2.07, *p* < 0.05) was significantly different. In contrast, by comparing in-degree and out-degree between the right HS and controls, we found that both the in-degree and out-degree of right MTG (*t* = 3.03, *p* < 0.005; *t* = 2.63, *p* < 0.02) were significantly different.

**Figure 4:**
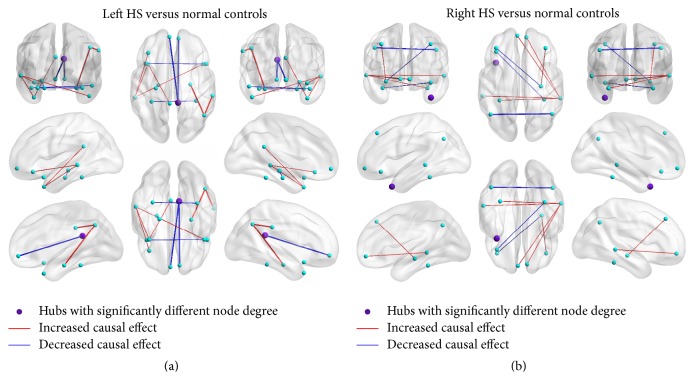
Changes of the driving effect between nodes in the semantic cognition network between the left HS, right HS, and normal controls. (i) The driving effect between nodes in ipsilateral structures increased and it decreased between nodes of contralateral structures in patients with left HS. (ii) Directional interaction weight changes between normal and patients with right HS seemed rather systematic, as an increased causal connectivity change was accompanied with a decreased intensity change and vice versa. (iii) The node degree of the right PCC was significantly different between patients with left HS and controls, and the node degree of the right MTG was significantly different between patients with right HS and controls.

Between-group analysis showed increased driving effect between nodes in ipsilateral structures and decreased driving effect between nodes of contralateral structures in left HS patients compared with normal controls. In detail, increased causal effects were found in the interactions from right AG to right parahippocampal gyrus, from right AG to right SMG, from left MTG to left parahippocampal gyrus, from left tpMTG to left PCC, and from left tpMTG to left MTG; decreased causal effects were found in the interactions from right PCC to left vmPFC, from right fusiform to left fusiform, and from left tpSTG to right tpSTG. There were also 3 exceptions where the causal effect from right vmPFC to right PCC and from left hippocampus to left parahippocampal gyrus decreased and where from right tpMTG to left MTG increased (see Figure 4(a)). By contrast, directional interaction weight changes between right HS patients and the controls seemed rather systematic. Increased interaction between ipsilateral ROIs originated from right vmPFC to right MTG; decreased intrahemisphere interaction originated from right hippocampus to right dmPFC. Increased interaction between contralateral ROIs originated from right AG to left AG, from left vmPFC to right MTG, and from right hippocampus to left dmPFC; decreased interhemisphere interaction originated from left fusiform to right MTG, from right fusiform to left IFG, and from right MTG to left MTG (see Figure 4(b)).

### 3.4. Correlation between Hippocampal LI and Epilepsy Onset Time, Duration, Frequency, and Strength of Causal Influence

There was significant correlation between hippocampus LI and changed path weights of effectively interconnected ROIs. The linking intensity from right AG to right parahippocampal gyrus in the left HS patients was negatively correlated with hippocampal LI (*r* = −0.56, *p* < 0.05), while the linking intensity from right AG to right SMG was positively correlated with hippocampal LI (*r* = −0.61, *p* < 0.05). No significant correlation was found between hippocampus LI and epilepsy onset time, duration, and frequency in both groups.

## 4. Discussion

To explore disrupted conceptualization in mTLE patients with HS, the current study focused on identifying structural and effective connectivity changes of the semantic cognition network. We found that the gray matter was significantly reduced in in both left and right HS patients. Even though the two hemispheres were equally damaged in mTLE patients with left HS, all the 7 regions that showed atrophy in the contralateral hemisphere were semantic cognition network components. Meanwhile, significant increased linking intensity changes between ipsilateral regions and decreased linking intensity changes between contralateral regions (particularly in the ATL area) were only found in the left mTLE group. The consistent anatomical and functional connectivity changes suggested that the breakdown of effective connectivity between left and right hemispheres, possibly caused by the severely damaged contralateral hemisphere, was the reason of more severe language impairment of left HS patients.

### 4.1. “Targeted Attacks” Affected the Contralateral Hemisphere of Left HS Patients Mostly

Previous quantitative MRI volumetric and voxel-based morphometry (VBM) studies have identified atrophy of the hippocampus [43] along with distributed abnormalities in neighboring and distant structures including the entorhinal cortex [43–45], parahippocampal gyrus [43], basal ganglia [46], lateral temporal cortex, frontal lobe, and cerebellum [47]. This distributed atrophy indicated influence of seizure propagation on the whole brain. Our findings were consistent with previous findings concerning the effect of epilepsy duration on gray matter volume in VBM studies [48, 49]. The altered topologies can be attributed to the seizure-dependent reinforcement of an epileptogenic configuration of the brain network.

Our findings also revealed different seizure propagation effects in the two patient groups. In the left HS patients, more regions in the whole brain, especially the dominant hemisphere, were injured. It was manifested that the left HS patients were more easily affected and may experience more serious hippocampal injuries, as their hippocampal LI varied more from normal controls. One theory about mechanisms underlying brain damage in mTLE hypothesizes that seizure propagation determines the distribution of damage [33]. Our findings matched such a theory, since left HS patients were more easily affected.

In addition, we specifically found that all the 7 atrophy regions in the right hemisphere in left HS patients were within the semantic cognition network. It indicated that left HS patients also displayed a higher vulnerability to seizure attacks in the potential compensatory semantic networks. We postulated that more serious anatomical changes in the left semantic cognition network and a disrupted compensatory mechanism in the contralateral hemisphere, which received “targeted attack” with higher vulnerability to disease, lead to more severe language impairment in the left HS patients.

### 4.2. Breakdown of Interhemisphere Connection, Especially in ATL, Induced More Severe Language Impairment in Left HS Patients

The results of the Granger causality analyses using functional ROIs showed no significantly different connection density among the left HS patients, right HS patients, and normal controls. However, node degree analysis revealed that hubs of patients' semantic cognition network changed. The right tpMTG was the only hub center in normal controls. Its importance was best manifested in the outflow condition. In contrast, the ATL was not the longer semantic cognition network hub in both left and right HS patients.

In particular, subregions of the ATLs (tpSTG and tpMTG) in the left and right hemispheres were causally affected by each other in normal controls. It was in stark contrast with semantic cognition networks in patients with left and right HS, in which connections between ATL subregions were all disrupted in left HS patients while only the bidirectional causal connection between left and right tpMTG remained in right HS patients. Patterson et al. [50] propose that bilateral anterior temporal lobes are amodal, abstract conceptual hubs that bind modality-specific properties, which are grounded in the sensory-motor system. Pobric et al. [51] used repetitive transcranial magnetic stimulation (rTMS) to disrupt neural processing temporarily in the left or right temporal poles and reported that rTMS disrupted semantic processing for words and pictures with the same degree. Their work illustrated that left and right anterior temporal lobes are critical in forming concepts of both words and pictures. Our findings suggested that even the residual weak interhemisphere interactions can sustain a relatively normal semantic network (in the right HS patients). Moreover, the integrating role of the semantic network hub in ATL (the right tpMTG) cannot be compensated by other region (such as the right PCC in the left HS patients), even though it may also be effectively connected with many areas in the network. It implicated that left and right ATLs functioned as a transmodal hub via mutual interconnections.

Changes in interregional functional coupling are thought to represent compensatory mechanisms secondary to structural pathology and seizure-related activity [52]. In terms of compensation strategies, patients with left HS showed that causal linking between nodes in ipsilateral structures increased, while causal effects between nodes of contralateral structures decreased. In contrast, patients with right HS showed a balanced change where the number of significantly increased interhemisphere and intrahemisphere causal interactions was the same as that of decreased interhemisphere and intrahemisphere causal interactions. The equal occurrence rate of altered effective changes may be a coincidence. However, it also indicates that if effective connections between ipsilateral and contralateral regions were damaged, patients with right HS were able to form a compensatory one to sustain a relatively normal semantic competence. It may owe to their less severe structural changes. In other words, the severe targeted atrophy in the contralateral hemisphere in patients with left HS caused disrupted interconnection between hemispheres and cannot be substituted for by intensified connection between regions in the same hemisphere. Thus, the breakdown of interhemisphere connections, especially those across left and right ATLs, leads to naming disability.

### 4.3. Hippocampal Sclerosis Was Accompanied with Reduced Causal Influence from the AG

The two significant correlations between hippocampus LI and the path weights of the semantic cognition network both originated from the right AG in left HS patients. Since gray matter of the right AG was also significantly reduced, the structural change of the right AG may influence the power flowing out from it. It was consistent with the conclusion that the AG occupies a position at the top of a processing hierarchy underlying concept retrieval and conceptual integration [12]. As impaired semantic control was associated with deregulated access to knowledge, patients may have difficulty directing activation towards the target and away from irrelevant prepotent associations [10].

## 5. Conclusions and Limitation

By comparing structural changes of left and right mTLE patients with healthy controls, the current study suggested that left HS patients had a higher vulnerability to seizure attacks, which may affect their compensation strategy. The interrupted effective connectivity between subregions of the ATL across hemispheres, which performs a unitary, homogeneous, transmodal representation for conceptual information, may be the primary reason why left HS patients displayed severe name finding difficulties but relevant good comprehension ability. In sum, our study revealed that the severe and targeted anatomical changes resulted in failed compensatory strategy in left HS patients, which was characterized by increased intrahemisphere causal interaction but decreased interhemisphere causal links.

The primary limitation in the current study is the small number of left and right HS patients. Our intention was to maintain uniformity across patients, even if it means sacrificing sample size. Incorporating patients with a loose standard may reduce detectability of group difference. However, it may also lead to no significant correlation between the magnitude of Granger causality interaction and patients' clinical data, such as disease onset time, duration, or seizure frequency.

## Figures and Tables

**Table 1:** Clinical and demographical data of the epilepsy patients.

Demographic	Left HS (*n* = 13)	Right HS (*n* = 11)
Age (mean ± SD, years)	27.3 ± 7.9	31.4 ± 11.6
Genders	5 males & 8 females	2 males & 9 females
Seizure frequency (times/week)	6.4 ± 6.4	8.4 ± 17.8
Epilepsy duration (years)	15.8 ± 8.6	15.2 ± 5.0
Seizures type	CPS	CPS
AEDs	CBZ, LTG, PHT	CBZ, LTG, PHT

CPS: complex partial seizures; AEDs: antiepileptic drugs; CBZ: carbamazepine; PHT: phenytoin; LTG: lamotrigine.

**Table 2:** Regions of GMV loss in left HS and right HS patients compared to healthy controls (FWE corrected, *p* < 0.05; cluster > 10 voxels).

Brain regions (AAL template)	Left HS (*n* = 13) voxels	Right HS (*n* = 11) voxels
**Frontal_Sup_Orb_L_05**	47	
**Frontal_Inf_Oper_L_11**	25	1
*Frontal_Inf_Orb_L_15*	39	
*Frontal_Inf_Orb_R_16*	141	
*Frontal_Mid_Orb_L_25*	11	
*Frontal_Mid_Orb_R_26*	31	
**Rectus_L_27**	82	
*Hippocampus_L_37*	75	
*ParaHippocampal_R_40*	57	
**Occipital_Sup_L_49**	146	
**Occipital_Mid_L_51**	368	
**Postcentral_L_57**	102	
**Parietal_Inf_L_61**	11	
*Angular_R_66*	61	
**Thalamus_R_78**		1
**Heschl_L_79**	65	
*Temporal_Pole_Sup_R_84*	329	
*Temporal_Mid_L_85*	315	
*Temporal_Mid_R_86*	351	1
*Temporal_Pole_Mid_R_88*	411	

Note: regions in italic are components of semantic cognition network; regions in bold are components of nonsemantic cognition network.
